# Spatial artifact detection improves the reproducibility of drug screening experiments

**DOI:** 10.1016/j.isci.2025.113470

**Published:** 2025-08-30

**Authors:** Aleksandr Ianevski, Kristen Nader, Swapnil Potdar, Alexandra Gorbonos, Filipp Ianevski, Ziaurrehman Tanoli, Jani Saarela, Tero Aittokallio

**Affiliations:** 1 Institute for Molecular Medicine Finland (FIMM), HiLIFE, University of Helsinki, Helsinki, Finland; 2 iCAN Digital Precision Cancer Medicine Flagship, University of Helsinki and Helsinki University Hospital, Helsinki, Finland; 3 Institute for Cancer Research, Department of Cancer Genetics, Oslo University Hospital, Oslo, Norway; 4 Oslo Centre for Biostatistics and Epidemiology (OCBE), Faculty of Medicine, University of Oslo, Oslo, Norway

**Keywords:** Natural sciences, Biological sciences, Bioinformatics, Pharmacoinformatics

## Abstract

Reliable and reproducible drug screening experiments are essential for drug discovery and personalized medicine. We demonstrate how systematic experimental errors in drug plates negatively impact data reproducibility, and that conventional quality control (QC) methods based on plate controls fail to detect these spatial errors. To address this limitation, we developed a control-independent QC approach that uses normalized residual fit error (NRFE) to identify systematic artifacts in drug screening experiments. Analysis of >100,000 duplicate measurements from the PRISM pharmacogenomic study revealed that NRFE-flagged experiments show 3-fold lower reproducibility among technical replicates. By integrating NRFE with QC methods to analyze 41,762 matched drug-cell line pairs between two datasets from the Genomics of Drug Sensitivity in Cancer project, we improved the cross-dataset correlation from 0.66 to 0.76. Available as an R package at https://github.com/IanevskiAleksandr/plateQC, plateQC provides a robust toolset for enhancing drug screening data reliability and consistency for basic research and translational applications.

## Highlights

• NRFE metric detects systematic spatial artifacts missed by existing quality control methods
• Integrating NRFE with existing methods improves cross-study correlation and data reliability
• NRFE-flagged low-quality plates exhibit 3-fold higher variability among technical replicates
• PlateQC R-package provides QC implementation and guidelines for drug screening workflows

## Graphical abstract

**Figure:**
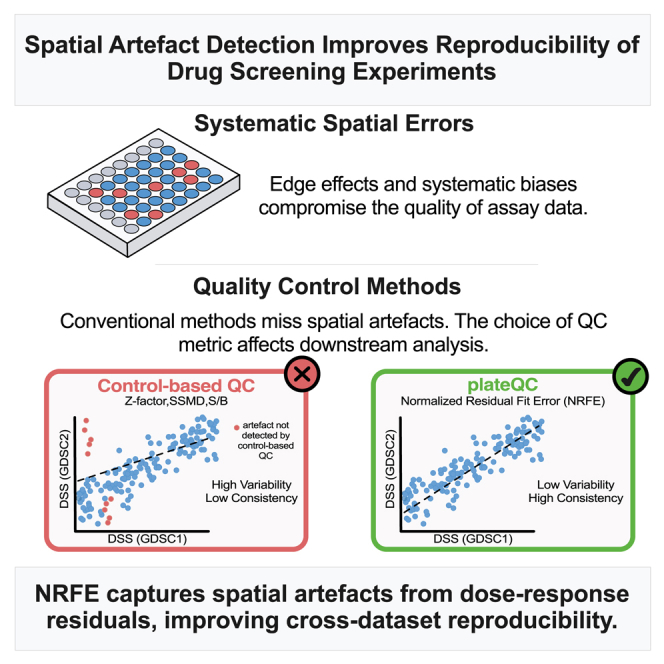

## Introduction

High-throughput screening (HTS) of cancer cell lines and patient-derived samples has become central to drug discovery and personalized medicine. Large-scale pharmacogenomic initiatives, such as Cancer Cell Line Encyclopedia (CCLE),^1^^,^^2^ Genomics of Drug Sensitivity in Cancer (GDSC),^3^^,^^4^ and Profiling Relative Inhibition Simultaneously in Mixtures (PRISM),^5^^,^^6^ have expanded our understanding of drug responses in diverse genetic backgrounds. Despite their broad impact, several studies have reported problems regarding inter-laboratory consistency and inter-replicate reproducibility of drug response measurements.^7^^,^^8^^,^^9^^,^^10^^,^^11^ These observations have led to valuable discussions about assay optimization strategies and best practices for cell-based drug response profiling, emphasizing the importance of robust validation approaches before translating preclinical findings.

Among various factors affecting drug screening data reproducibility,^12^ one important consideration is the experimental data quality. Quality control in HTS drug experiments traditionally relies on simple plate control-based metrics and universal cutoff values, adhering to widely accepted standards such as Z-prime/Z-factor (Z' > 0.5),^13^ Strictly Standardized Mean Difference (SSMD >2),^5^^,^^14^ and signal-to-background ratio (S/B > 5)^13^^,^^15^ as the primary plate quality indicators even in the recent HTS studies.^4^^,^^16^^,^^17^^,^^18^^,^^19^^,^^20^ While these QC approaches have provided valuable and straightforward quality assessment for decades of HTS, they are fundamentally limited in their ability to detect many critical issues that can compromise experimental data quality. Control wells, by their nature, can only assess a fraction of the plate spatial area, and therefore, the control-based QC metrics cannot capture systematic errors that affect the drug wells.^21^

The inherent challenges in HTS quality assessment stem from multiple factors. First, compound-specific issues - such as drug precipitation,^22^ stability changes during storage or assay conditions,^23^ carryover between wells during liquid handling^24^ or interference with assay readouts^25^ – can significantly impact data quality, even when control wells appear adequate. Second, plate-specific artifacts - including evaporation gradients,^26^ systematic errors in pipetting,^27^ and temperature-induced drift^28^^,^^29^^,^^30^ - can create spatial patterns of variability that may affect control and sample wells differently or occur in regions not covered by the controls. Third, position-dependent effects - such as striping or edge-well evaporation^31^^,^^32^ that lead to artificially high drug concentrations, or location-specific aggregation - introduce systematic errors that the control-based metrics fail to detect.

These quality issues often remain undetected when using the existing QC approaches and can therefore significantly impact the screening readouts and downstream analyses. Even plates passing traditional control-based quality metrics may still harbor systematic spatial errors that affect drug response measurements and dose-response curve fitting.^31^^,^^33^ As we demonstrate herein, these undetected errors significantly impact reproducibility, and their removal leads to marked improvements both in technical replicates and cross-dataset correlation. Importantly, these spatial errors can lead to unreliable drug response quantifications using response metrics such as AUC or IC50 - particularly when spatial artifacts coincide with compound concentration patterns. In large-scale screens, such issues may cause inconsistent results between replicates, compromise the identification of true hits, and ultimately misdirect follow-up studies. These inconsistencies have direct consequences on the consistency of preclinical drug profiling results across different laboratories.^34^^,^^35^^,^^36^^,^^37^^,^^38^

To address these challenges, we developed a quality assessment approach based on normalized residual fit error (NRFE), which evaluates plate quality directly from drug-treated wells rather than relying on control wells. By analyzing deviations between the observed and fitted response values, while accounting for the variance structure of dose-response data, the method identifies systematic spatial errors in the drug wells that the control-based metrics fail to detect. Analysis of four large-scale pharmacogenomic datasets (GDSC1, GDSC2, PRISM, and FIMM) demonstrates that the NRFE metric complements traditional control-based approaches; while control-based metrics excel at detecting assay-wide technical issues, NRFE captures drug-specific and position-dependent spatial artifacts. The integration of these orthogonal approaches therefore substantially improves our ability to identify unreliable drug response data, and enhance the quality, reproducibility and consistency of drug screening experiments.

## Results

### Normalized residual fit error detects systematic plate artifacts

**Figure 1:**
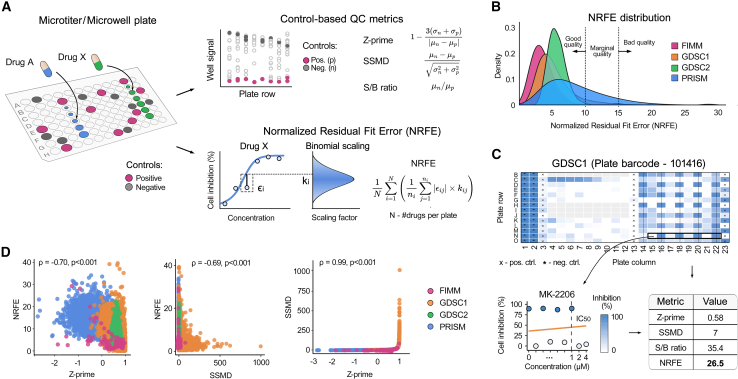
NRFE provides complementary quality assessment to traditional control-based metrics (A) Calculation principles for drug screening plate quality control (QC) metrics: traditional control-based metrics (Z-prime, SSMD, S/B) are based on within plate positive and negative control wells (top), and NRFE approach makes use of dose-response curves from compound-containing wells (bottom). See STAR Methods for the mathematical definitions of the metrics. (B) NRFE distributions across large-scale pharmacogenomic screens show relatively consistent quality baselines, with modal values of 2.8 (FIMM, *n* = 1,044 plates), 3.9 (GDSC1, *n* = 17,961), 5.1 (GDSC2, *n* = 11,440), and 5.9 (PRISM, *n* = 49,545). (C) Top panel: an example heatmap of the plate 101416 from the GDSC1 dataset shows column-wise striping artifacts on the right side of the plate. Bottom panels: an example of a compromised dose-response curve for compound MK-2206 (left) from plate 101416 that passed traditional quality metrics but was flagged by its extremely high NRFE value (right). (D) Correlation analysis between the quality metrics revealed a strong Spearman correlation between Z-prime and SSMD, but only a moderate negative correlation with NRFE (*p* < 0.001 for all comparisons, permutation test). Each point corresponds to a separate plate in the datasets.

To systematically evaluate experimental plate quality in HTS experiments, we first examined traditional control-based metrics and identified their limitations. While metrics such as Z-prime (Z′), SSMD, and signal-to-background ratio (S/B) have been widely adopted as industry standards, they rely solely on control wells to assess plate quality. Z-prime evaluates separation between positive and negative controls using means and standard deviations, SSMD quantifies the normalized difference between controls, and S/B measures the ratio of mean control signals (Figure 1A, right top panel). Recognizing the need to detect artifacts in drug-containing wells, we developed the Normalized Residual Fit Error (NRFE) metric, which is based on deviations between the observed and fitted values in dose-response curves across all compound wells, and applies a binomial scaling factor to account for response-dependent variance (see STAR Methods, Figure 1A, right bottom panel).

To establish robust quality control thresholds for NRFE, we analyzed its distribution across 79,990 drug plates from four large-scale pharmacogenomic datasets (GDSC1, GDSC2, PRISM, and FIMM; Figure 1B; Figure S1). These datasets were selected as they provide both dose-response measurements and plate location information required for systematic quality assessment. While GDSC1, GDSC2, and FIMM showed similar distributions, PRISM exhibited systematically higher NRFE values, likely due to its distinct experimental setup that uses a pooled-cell screening format, longer treatment time (120h vs. 72h), and luminex-based readout rather than standard cell viability assays. Notably, PRISM also showed substantially worse performance in the traditional control-based metrics (Z-prime and SSMD; Figure S1).

In the better-quality datasets, values exceeding three standard deviations corresponded to NRFE thresholds of approximately 10 in GDSC1 and 15 in both GDSC2 and FIMM datasets (Figure 1B). These statistically derived thresholds were validated using previously identified low-quality plates from internal FIMM screening data (see STAR Methods), which led to NRFE values predominantly above 15 (Figure S1A). Based on this convergence of statistical analysis and internal validation, we defined three quality tiers: NRFE >15, indicating low quality and requiring exclusion or careful review, 10–15 indicating borderline quality and requiring additional scrutiny, and NRFE <10 indicating acceptable quality. These empirically validated NRFE thresholds complement the established control-based cut-offs, Z-prime (>0.5) and SSMD (>2).

The practical utility of NRFE became evident in its ability to identify problematic plates that passed the control-based quality control. For instance, plate 101416 from the GDSC1 dataset exhibited pronounced column-wise striping in the right half of the plate (Figure 1C). This spatial pattern, likely arising from liquid handling irregularities, severely affected the dose-response relationships of multiple compounds, including MK-2206, which showed irregular, jumpy dose responses that deviated from the expected sigmoid behavior (Figure 1C, bottom panel). Despite these clear artifacts, traditional metrics indicated an acceptable quality (Z-prime = 0.58, SSMD = 7, S/B = 35.4), while an extremely high NRFE of 26.5 flagged the systematic quality issues (Figure 1C, right-bottom panel). Additional examples of NRFE detecting systematic artifacts missed by the control-based metrics are shown in Figure S2.

Interestingly, when examining correlations between the QC metrics in these datasets, we found that S/B, which relies solely on averaged control signals without considering their variability, showed the weakest correlations with the other metrics (|ρ|<0.2, *p* < 0.001, Figure S3). In contrast, Z-prime and SSMD were highly correlated (ρ = 0.99, *p* < 0.001), indicating that they capture similar quality aspects, while NRFE showed only a moderate negative correlation with both metrics (Z-prime: ρ = −0.70, SSMD: ρ = −0.69, *p* < 0.001) (Figure 1D). This lower yet significant correlation indicates that NRFE considers distinct quality aspects, compared to the control-based metrics, supporting its use as a complementary approach for detecting systematic errors in drug-response assays.

### Normalized residual fit error predicts the technical reproducibility of drug response measurements

**Figure 2:**
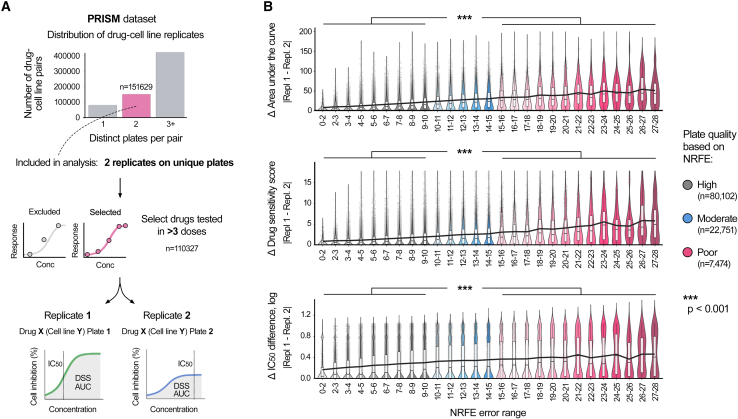
NRFE identifies drug response measurements with reduced technical reproducibility (A) Overview of the PRISM dataset reproducibility analysis workflow: from more than 500,000 drug-cell line combinations, we identified 151,629 pairs with measurements on exactly two distinct plates, of which 110,327 had sufficiently many concentration points (>3 doses) for reliable curve fitting. (B) Impact of NRFE plate quality on the measurement reproducibility. Drug-cell line pairs were categorized by their plate NRFE values into high (gray, *n* = 80,102), moderate (blue, *n* = 22,751), and poor (red, *n* = 7,474) quality categories. Black trend lines indicate the median values across NRFE categories, showing increasing variability between replicate measurements as a function of NRFE level for three drug response metrics: area under the curve (AUC), drug sensitivity score (DSS), and half maximal inhibitory concentration (IC50). The boxplots show the median (central line), 25th and 75th percentiles (box edges), and the range within 1.5 times the interquartile range from the box (whiskers). Statistical significance (p values) was calculated using the Wilcoxon test; ∗∗∗, p < 0.001.

We next examined whether plates with elevated NRFE levels exhibit reduced reproducibility in drug response measurements. For this analysis, we focused on the PRISM dataset, which, despite showing lower quality metrics overall, provided an ideal testbed with over 500,000 drug-cell line combinations tested across multiple plates. Within this extensive dataset, we identified 151,629 drug-cell line pairs with independent measurements on exactly two unique plates (Figure 2A). To ensure reliable dose-response curve fitting, we further subselected 110,327 cases where the drugs were tested across more than three concentrations.

A striking pattern emerged when we categorized the drug-cell line measurements according to their plate NRFE values into three quality categories: high (NRFE< 10, *n* = 80,102), moderate (10≤NRFE≤15, *n* = 22,751), and poor (NRFE>15, *n* = 7,474), Figure 2B. By comparing the replicate measurements between plates of different quality categories, we found that the pairs where at least one replicate came from a poor-quality plate showed substantially worse reproducibility, compared to the high-quality plates (*p* < 0.001, Wilcoxon test), with black trend lines emphasizing the consistent relationship between increasing NRFE values and measurement variability (Figure 2B). This effect was consistent across three commonly used drug response metrics: area under the curve (AUC, overall drug sensitivity), drug sensitivity score (DSS, normalized AUC),^39^ and half maximal inhibitory concentration (IC_50_, drug potency) values. For example, replicate measurements from high and moderate quality plates showed AUC differences of 11.8 on average, while those involving poor-quality plates exhibited average differences of 29.8 - a 3-fold increase in response variability.

Notably, the poor-quality measurements comprised only 6.8% of the whole PRISM dataset, suggesting an opportunity to substantially improve data reliability through targeted removal of a small fraction of the most problematic plates. This finding has important implications for large-scale drug screening campaigns, which rely on maintaining broad coverage while ensuring sufficient data quality.

### Integration of quality control metrics improves cross-dataset consistency

**Figure 3:**
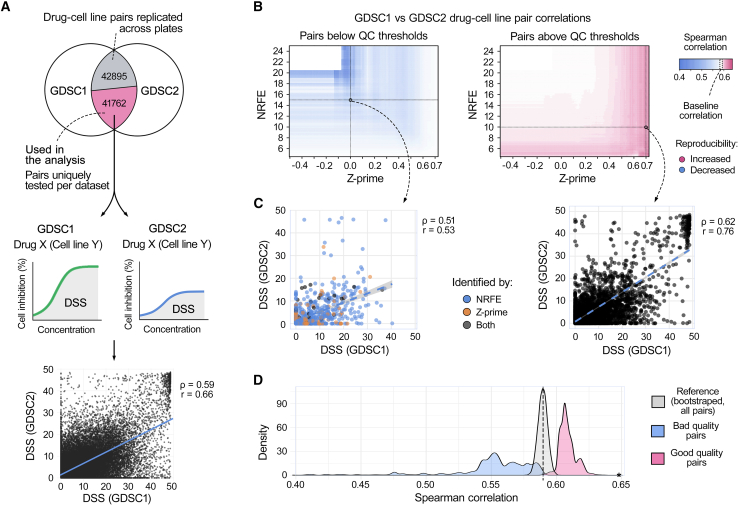
Quality control metrics contribute to drug response consistency across datasets (A) Analysis workflow: Venn diagram of 84,657 matching drug-cell line pairs between GDSC1 and GDSC2 datasets, with 41,762 pairs appearing exactly once in each dataset, enabling direct plate-level quality assessment (top). Baseline correlation of drug sensitivity scores (DSS) between datasets shows moderate consistency (Spearman ρ = 0.59, Pearson r = 0.66) (bottom). (B) Impact of quality thresholds on consistency shown as correlation heatmaps across Z-prime (−0.5 to 0.75) and NRFE (4–25) ranges. Left: correlations for the pairs with poor quality metrics (Z-prime below or NRFE above threshold in either dataset), with dotted lines indicating quality thresholds (NRFE = 15, Z-prime = 0). Right: correlations for the pairs with good quality metrics (Z-prime above and NRFE below threshold in both datasets), with dotted lines marking quality thresholds (NRFE = 10, Z-prime = 0.7). White indicates the baseline correlation (ρ = 0.59), with red and blue showing increased and decreased consistency, respectively. (C) Impact of combined quality metrics on consistency: the pairs from poor-quality plates (NRFE >15, Z-prime <0) show reduced correlation (ρ = 0.51, r = 0.53), while the pairs from high-quality plates (Z-prime >0.7, NRFE <10) show improved correlation (ρ = 0.62, r = 0.76), compared to the baseline (ρ = 0.59, r = 0.66). (D) Statistical validation using bootstrap sampling to establish a reference baseline distribution, compared with distributions from poor-quality plates (Z-prime <0 or NRFE >15) and high-quality plates (Z-prime >0.5 and NRFE <10). We systematically explored various quality thresholds by calculating correlations at 0.02 intervals across the full range of Z-prime (−0.5 to 0.75) and NRFE (4–25) values. The asterisk on x axis indicates top 2% highest-quality plates (NRFE <6.36, Z-prime >0.73, ρ = 0.65, r = 0.80). All differences were significant at *p* < 0.001 (Kolmogorov-Smirnov test).

After establishing the ability of NRFE to identify less reproducible measurements within the PRISM dataset, we next investigated whether combining multiple quality metrics could further improve consistency across different studies. For this analysis, we compared the drug response measurements between the GDSC1 and GDSC2 datasets. From 84,657 matching drug-cell line pairs between the two datasets, we focused our analysis on 41,762 pairs that appeared exactly once in each dataset, enabling direct plate-level quality assessment (Figure 3A). To quantify drug response consistently, we calculated drug sensitivity scores (DSS), a normalized area under the dose-response curve metric. Initial analysis of all matched pairs revealed a moderate cross-dataset correlation (Spearman ρ = 0.59 and Pearson r = 0.66; Figure 3A, bottom panel), providing a baseline for evaluating quality-dependent drug response data consistency.

To systematically evaluate the combined impact of different quality metrics on the consistency, we stratified the matched drug-cell line pairs based on both their plate Z-prime (range −0.5 to 0.75) and NRFE values (range 4–25). For each threshold combination, we calculated Spearman correlations separately for the drug-cell line pairs with poor quality control metrics (Z-prime below the threshold or NRFE above the threshold in either dataset) and good quality control metrics (Z-prime above the threshold and NRFE below the threshold in both datasets). The visualization of these correlations as heatmaps demonstrated that the drug-cell line pairs derived from plates with poor quality metrics showed decreased consistency, while pairs with acceptable quality metrics exhibited improved consistency, when compared to the baseline correlation of ρ = 0.59 (Figure 3B).

The added value of combining the quality metrics became evident when examining specific threshold combinations across the dataset of 41,762 drug-cell line pairs (Figure 3C). For example, drug-cell line pairs from the poor-quality plates with both high NRFE and low Z-prime values (NRFE >15 and Z-prime <0) showed a reduced cross-dataset correlation (ρ = 0.51, r = 0.53), while pairs from the plates with excellent quality based on both metrics (Z-prime >0.7 and NRFE <10) demonstrated an increased correlation (ρ = 0.62 and r = 0.76), when comparing to the baseline correlation (ρ = 0.59 and r = 0.66). These improvements in drug response data consistency across large-scale pharmacogenomic datasets demonstrate how integrating multiple quality metrics can systematically identify more reliable drug response measurements.

To statistically validate these findings, we used a bootstrap sampling of the matched drug-cell line pairs to establish a reference distribution for the baseline consistency (Figure 3D). Two quality groups were then compared against the reference distribution: lower-quality pairs (defined by either Z-prime <0 or NRFE >15) and high-quality pairs (defined by both Z-prime >0.5 and NRFE <10). Both groups showed significant differences compared to the reference distribution (*p* < 0.001, Kolmogorov-Smirnov test; Figure 3D). Further analysis of more stringent thresholds revealed that the top 2% highest-quality plates (NRFE <6.36 and Z-prime >0.73) achieved even stronger consistency, with correlations of ρ = 0.65 and r = 0.80 (Figure 3D, asterisk). These results demonstrate how combining traditional control-based metrics with NRFE provides a robust framework for identifying reliable drug response measurements across drug screening studies.

## Discussion

Preclinical drug response reproducibility remains one of the most critical challenges in drug development and personalized medicine studies, as non-reproducible preclinical findings compromise therapeutic success in follow-up studies and clinical trials, with oncology having the highest failure rate compared to other therapeutic areas.^33^^,^^34^ Our comprehensive analysis reveals that this reproducibility challenge stems not only from technical variability in drug screening assays but also from fundamental limitations in the current quality assessment approaches. Traditional control-based metrics, while valuable for detecting assay-wide technical issues, are inherently limited by their focus on control wells, potentially missing systematic errors that affect the drug response measurements.

The NRFE metric overcomes these limitations by assessing systematic spatial errors directly in drug-treated wells, hence providing a complementary approach to traditional quality control. Our analysis of the relationships between quality metrics revealed their distinct roles - while Z-prime and SSMD showed a strong correlation (ρ = 0.99) in detecting assay-wide issues, NRFE showed a moderate negative correlation with these metrics (ρ ≈ −0.7; Figure 1D), indicating its ability to capture different quality aspects. As shown by the representative examples in Figure 1C, these metrics identify distinct types of experimental artifacts, indicating their complementary roles in the comprehensive quality assessment of drug response measurements. The weak correlations of signal-to-background ratio with the other metrics (|ρ| < 0.2, Figure S3) question its continued use in screening protocols, particularly given that it ignores the control well variability.^40^

The NRFE metric offers several practical advantages for drug screening workflows. It requires only percent inhibition (or viability) values, enabling quality assessment even without raw plate reader data. However, since NRFE relies on dose-response curve fitting, it is specifically designed for multi-dose assays, highlighting the continued importance of control-based metrics for single-dose screens. Our curve fitting implementation includes optimizations for extreme cases commonly encountered in drug screening, with robust fallbacks for highly potent or inactive compounds that were originally developed in our Breeze platform.^15^ However, compounds exhibiting non-sigmoid responses, such as bell-shaped curves from high-concentration cytotoxicity, may result in elevated residuals. While NRFE’s plate-level averaging makes it robust to occasional poor curve fits, plates containing many compounds with non-sigmoid responses could potentially be incorrectly flagged as having poor quality and should be manually inspected to distinguish between systematic spatial artifacts and compound-specific curve fitting issues.

To facilitate the adoption of this integrated quality control approach, we developed the plateQC R package that implements both control-based and drug-based metrics with interactive visualizations. The R package provides comprehensive tools for plate-level quality assessment, including the automated detection of spatial artifacts, interactive visualization of dose-response curves, and statistical summaries of quality metrics. We recommend examining plates that fall below the established quality thresholds (see Figure S2). Based on these thresholds, we recommend the following actions: plates with NRFE <10 and passing other QC metrics can be confidently used for analysis; those with NRFE 10–15 should undergo visual inspection and manual review before inclusion; and plates with NRFE >15 should be excluded and repeated, if possible, due to systematic spatial artifacts. The primary validation of these thresholds originated from our PRISM reproducibility analysis, where plates with NRFE >15 demonstrated significantly worse technical reproducibility between replicates, while representing only 6.8% of the dataset, indicating appropriately stringent criteria without excessive data loss. Similar to other QC metrics, NRFE threshold selection involves empirical optimization - like the Z-prime recommendations that vary across applications (with some researchers accepting values as low as −0.5 in challenging assays, even though not recommended) - NRFE thresholds may require adjustment based on the specific assay conditions and data quality requirements.

Analysis of four large-scale pharmacogenomic datasets (GDSC1, GDSC2, PRISM, and FIMM) demonstrates the broad applicability of NRFE approach across diverse screening platforms, assays, and protocols. Notably, the integration of NRFE with traditional metrics, such as Z-prime, enabled the identification of more reliable measurements in both technical replicates and cross-dataset comparisons, hence further improving data reproducibility and consistency. The substantial improvement in the cross-dataset correlation between GDSC1 and GDSC2 after filtering by quality metrics (from 0.66 to 0.76) is particularly noteworthy, as these drug testing datasets employed different experimental protocols. This cross-protocol validation demonstrates that NRFE and Z-prime quality metrics successfully identified higher-quality measurements that remain consistent, even when the experimental conditions change, strengthening confidence in NRFE’s ability to detect fundamental quality issues rather than protocol-specific artifacts. Since these large-scale datasets underpin numerous preclinical discovery and computational modeling efforts, improved quality control is expected to enhance the reliability of the downstream analyses and reduce the costs throughout the drug development process. The improved reliability and translatability of the preclinical findings will accelerate the production of new and desperately needed therapies for many diseases.

In conclusion, this work establishes a comprehensive framework for quality control in high-throughput drug screening by combining control-based and drug-specific error detection approaches. While multiple factors influence experimental reproducibility, the systematic identification of reliable measurements represents an important step toward more robust preclinical data generation.

### Limitations of the study

The NRFE metric was designed for multi-dose drug screening assays, and therefore, it cannot evaluate the quality of single-dose screens, limiting its application in certain high-throughput formats. This work focused only on cell viability-based drug response readouts from large-scale pharmacogenomics studies. The applicability to other screening readouts and assays, such as reporter-based assays or biochemical screens, remains to be validated. Compounds exhibiting legitimate non-sigmoid dose-response relationships, such as bell-shaped curves from cytotoxicity, may result in elevated NRFE values, potentially requiring manual inspection to distinguish between systematic spatial artifacts and compound-specific response patterns.

## Resource availability

### Lead contact

Further information, requests, or inquiries should be directed to and will be fulfilled by the Lead contact, Tero Aittokallio, tero.aittokallio@helsinki.fi.

### Materials availability

This study did not generate new unique reagents.

### Data and code availability

• Data

The PRISM, GDSC1 and GDSC2 datasets were accessed through the DepMap: https://depmap.org/portal/data_page/?tab=customDownloads; accession codes listed in the key resources table. The FIMM dataset was accessed through PharmacoDB: https://pharmacodb.ca/; key resources table and extended with an internal dataset of 16 poor-quality plates based on visual analysis. The internal dataset originates from patient-derived cell screening and in-house hospital and commercial projects, and is available from the lead contact upon reasonable request. Access to these internal data will require authorization by governing bodies and analysis via a secure analysis environment at the Helsinki University Hospital datalake.

• Code

The *plateQC* R package implements all quality control metrics described in this study, including interactive plate visualizations and robust outlier detection options. The R package is freely available at GitHub: https://github.com/IanevskiAleksandr/plateQC and archived in Zenodo repository: https://doi.org/10.5281/zenodo.16737294.^41^ Analysis scripts used to generate the results and figures are available from the corresponding author upon reasonable request.

## Acknowledgments

We thank the DDCB core facility (FIMM HTB unit) supported by the University of Helsinki and Biocenter Finland. Funding support: AI: Ida Montin Foundation grant. TA: 10.13039/501100002341Research Council of Finland (grants 340141, 344698, 367855); the 10.13039/501100006383Cancer Society of Finland, the 10.13039/100008730Norwegian Cancer Society (grants 216104 and 273810), Norwegian Health Authority South-East (grants 2020026 and 2023105), the 10.13039/501100006306Sigrid Jusélius Foundation, and iCAN – Digital Precision Cancer Medicine Flagship (iCAN-MULTIDRUG). This work was supported by the REMEDi4ALL project, which has received funding from the European Union’s Horizon Europe research and innovation programme under grant agreement No 101057442. Views and opinions expressed are those of the author(s) only and do not necessarily reflect those of the European Union, who cannot be held responsible for them.

## Author contributions

Equal contributors (A.I., K.N.) conceived the study, developed the NRFE methodology, designed and implemented the plateQC R package, performed statistical analyses, created visualizations, and wrote the article. S.P. and J.S. contributed to software testing and article writing. A.G. performed data curation and quality control analyses and helped with the figure drafts. F.I. contributed to statistical methodology, data analysis, and article writing. Z.T. contributed to data processing and article writing. T.A. supervised the project, contributed to conceptualization and methodology, provided funding, and contributed to writing, review, and editing.

## Declaration of interests

The authors declare no competing interests.

## STAR★Methods

### Key resources table

**Table:** 

REAGENT or RESOURCE	SOURCE	IDENTIFIER
**Deposited data**		

GDSC1 drug screening data	DepMap Portal	https://depmap.org/portal/data_page/?tab=customDownloads
GDSC2 drug screening data	DepMap Portal	https://depmap.org/portal/data_page/?tab=customDownloads
PRISM drug screening data	DepMap Portal	https://depmap.org/portal/data_page/?tab=customDownloads
FIMM drug screening data	PharmacoDB	https://pharmacodb.ca/

**Software and algorithms**		

R v.4.3.1 statistical software	R Core Team	https://www.r-project.org/
plateQC v1.0.0 R package	This paper	https://github.com/IanevskiAleksandr/plateQC
boot v.1.3-31 R package	Canty and Ripley	https://CRAN.R-project.org/package=boot
drc v.3.0-1 R package	Ritz et al.	https://CRAN.R-project.org/package=drc
ggplot2 v.3.5.2 R package	Wickham et al.	https://CRAN.R-project.org/package=ggplot2

### Experimental model and study participant details

This study did not generate or use experimental models such as cell lines, animals, plants, microbes or primary cultures. Instead, all analyses were conducted using publicly available pharmacogenomic datasets and one anonymized internal dataset, as detailed below.

• Public Datasets (PRISM, GDSC, FIMM)

The PRISM, GDSC1, and GDSC2 datasets were accessed from the DepMap portal, and the FIMM dataset from PharmacoDB. These datasets include human cancer cell line drug response measurements. Detailed information on the cell line origin, culture conditions, authentication (STR profiling), mycoplasma testing, and participant details (age, sex, ethnicity, and so forth) are provided in the original publications describing these datasets.^3^^,^^4^^,^^5^^,^^6^^,^^42^

• FIMM Internal Dataset

In addition to the public FIMM dataset, we analyzed an anonymized internal dataset derived from patient-derived *ex vivo* drug sensitivity studies conducted at the Institute for Molecular Medicine Finland (FIMM). The dataset originates from in-house projects. No stratification into experimental groups was performed for this study. As the data were fully anonymized, individual-level information such as age, gender, ethnicity, and clinical details were not available.

• Consideration of Sex and Gender

This study analyzed large-scale drug sensitivity datasets in an aggregated manner. Metadata on sex, gender, and related demographic variables were not analyzed here, and therefore their potential influences on the results could not be assessed.

### Method details

#### Calculation of quality control metrics

Traditional control-based metrics (Z-prime, SSMD, and S/B) are calculated using positive and negative control wells. The Z-prime factor (also known as Z′ or Z-factor), being the most widely adopted industry standard, quantifies the separation between positive (p) and negative (n) control wells, calculated as Z′ = 1 - (3σ_p_ + 3σ_n_)/|μ_p_ - μ_n_|, where σ and μ represent standard deviation and mean, respectively, over the plate controls. Strictly Standardized Mean Difference (SSMD) is computed as (μ_p_ - μ_n_)/√(σ_p_^2^ + σ_n_^2^). Signal-to-background ratio is calculated as S/B = μ_p_/μ_n_.

The Normalized Residual Fit Error (NRFE) is defined based on normalized residuals from a four-parameter log-logistic (LL4) model fit. We chose the widely used LL4 model in the present study, but we note that NRFE can also be calculated based on other dose-response functions. The LL4 model was defined as f(x) = d + (a - d)/(1 + (x/c)^b^), where a is the minimum asymptote, d is the maximum asymptote, b is the slope, c is the inflection point (IC_50_), and x is the drug concentration. The residual errors εᵢⱼ at each concentration point j was calculated as |yᵢⱼ - f(xᵢⱼ)|, where yᵢⱼ is the observed response for drug *i* at concentration point *j*. NRFE is then computed as (1/N) × Σᵢ_=1_ᴺ ((1/nᵢ) × Σⱼ_=1_^ni^ |εᵢⱼ × kᵢⱼ|), where *N* is the number of drug wells per plate, nᵢ is the number of concentrations for drug *i*, and *k*ᵢⱼ is the binomial scaling factor. When technical replicates are present at the same concentration, each replicate contributes independently to the dose-response curve fitting, and the residual errors are calculated for each individual measurement rather than using averaged values. The scaling factor kᵢⱼ is calculated as 1 + (p × (1-p)/0.25), where p is the fitted response proportion (f(xᵢⱼ)/100). This scaling factor adjusts for the weight of residuals based on their position in the dose-response curve, with the maximal weight at 50% response. Quality thresholds were established as NRFE > 15 for low quality requiring review, 10-15 for marginal quality needing scrutiny, and NRFE <10 for acceptable quality.

### Quantification and statistical analysis

#### Statistical assessment of correlation differences

To assess whether the correlations differed between quality-stratified groups, we first established a reference distribution of expected correlations by bootstrapping the original drug sensitivity scores (DSSs) (10,000 resamples with replacement). For each resample, we calculated the Spearman correlation between GDSC1 and GDSC2 dataset DSS scores, creating a distribution of background correlations under typical conditions. We then compared this reference distribution against the correlation distributions from two quality groups: low-quality plates (defined by NRFE > 15 or Z-prime < 0) and high-quality plates (NRFE < 10 and Z-prime > 0.5). Statistical significance was assessed using the two-sample Kolmogorov-Smirnov tests, which evaluates the null hypothesis that the two samples come from the same distribution. Effect sizes were calculated as standardized mean differences between each group and the reference distribution was, computed as the difference in means divided by the pooled standard deviation.

#### Determining NRFE threshold

NRFE thresholds were established using distribution analysis across 79,990 drug plates from four datasets analysed in this work. Values exceeding three standard deviations from the mean corresponded to NRFE thresholds of 10 (GDSC1) and 15 (GDSC2 and FIMM). These thresholds were validated against 16 visually identified poor quality plates from an internal FIMM dataset. The plate results were defined as acceptable (NRFE < 10), borderline (10-15) and poor-quality requiring exclusion or repetition (NRFE > 15).

#### Software and statistical packages

All statistical analyses were performed in R version 4.2.3. Statistical computations used the stats package for correlation analysis and Kolmogorov-Smirnov tests. Bootstrap analysis utilized the boot^42^ package (v. 1.3-28.1). Dose-response curve fitting was performed using the drc package (v. 3.0-1). Data visualization was created using ggplot2 (v. 3.5.2). The plateQC R-package implements advanced quality control metrics calculation (NRFE, Z-factor, robust Z-prime, SSMD, signal-to-background ratio,) and interactive plate visualization (inhibition heatmaps, residual error heatmaps, row-wise distribution plots).
