# Supplementary material for: Spatial artifact detection improves the reproducibility of drug screening experiments
Source: iScience. 2025 Aug 30;28(10):113470. doi: 10.1016/j.isci.2025.113470 (PMC12570324; doi:10.1016/j.isci.2025.113470)
Supplement: Document S1. Figures S1–S3 [file mmc1.pdf]

iScience, Volume *28*

Supplemental information

Spatial artifact detection improves

the reproducibility of drug screening experiments

Aleksandr Ianevski, Kristen Nader, Swapnil Potdar, Alexandra Gorbonos, Filipp Ianevski, Ziaurrehman Tanoli, Jani Saarela, and Tero Aittokallio

Supplementary Information for

Spatial artefact detection improves reproducibility of drug screening experiments

Aleksandr Ianevski, Kristen Nader, Swapnil Potdar, Alexandra Gorbonos, Filipp Ianevski, Ziaurrehman Tanoli, Jani Saarela, Tero Aittokallio

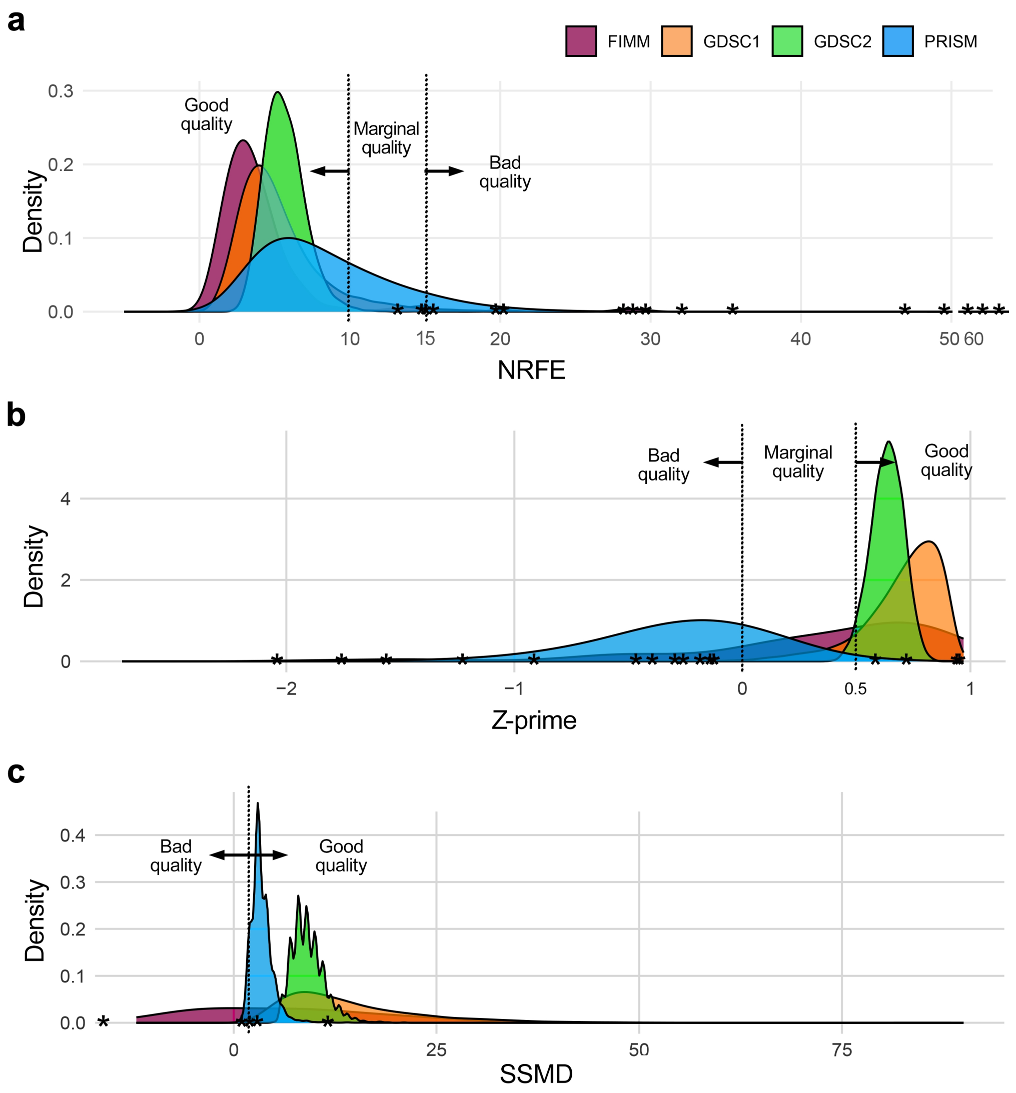

Supplementary Figure 1 | Quality metric distributions across four pharmacogenomic screening datasets. (**a**) Normalized Relative Fit Error (NRFE) distributions showing the quality thresholds: values >15 indicate poor quality plates requiring exclusion or detailed review, 10-15 indicate marginal quality needing additional scrutiny, and <10 represent acceptable quality plates. The stars indicate 16 manually curated poor-quality plates from the in-house FIMM dataset that were visually identified to contain systematic artifacts. (**b**) Z-prime factor distributions demonstrating assay separation between positive and negative controls across all plates. Z-prime values >0.5 indicate excellent assay quality, 0-0.5 indicate marginal quality requiring additional scrutiny, and Z'<0 indicate poor separation between controls necessitating assay optimization or plate exclusion. (**c**) Strictly Standardized Mean Difference (SSMD) distributions measuring the effect size between control populations. SSMD >2 indicates good quality and SSMD <2 poor quality necessitating careful evaluation or exclusion.

a

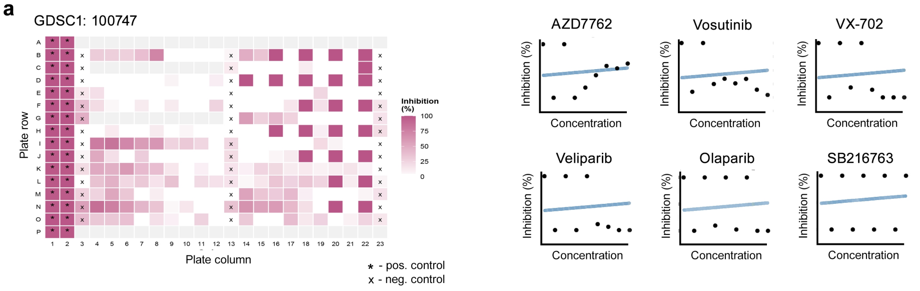

Metric	Value
Z-prime	***0.09**
SSMD	7
S/B ratio	3
NRFE	***30**

b

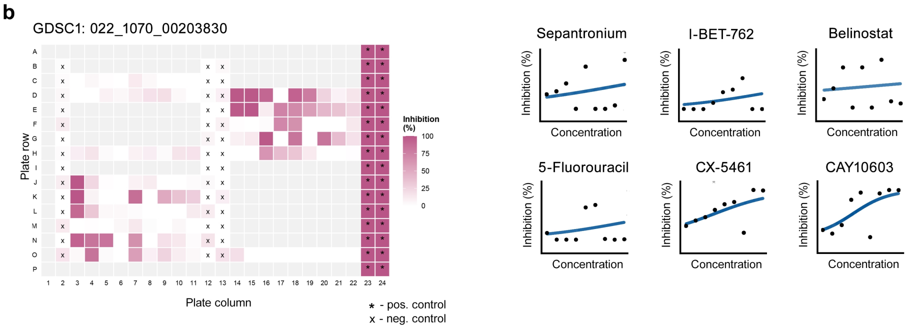

Metric	Value
Z-prime	0.73
SSMD	11
S/B ratio	16.4
NRFE	***25.1**

c

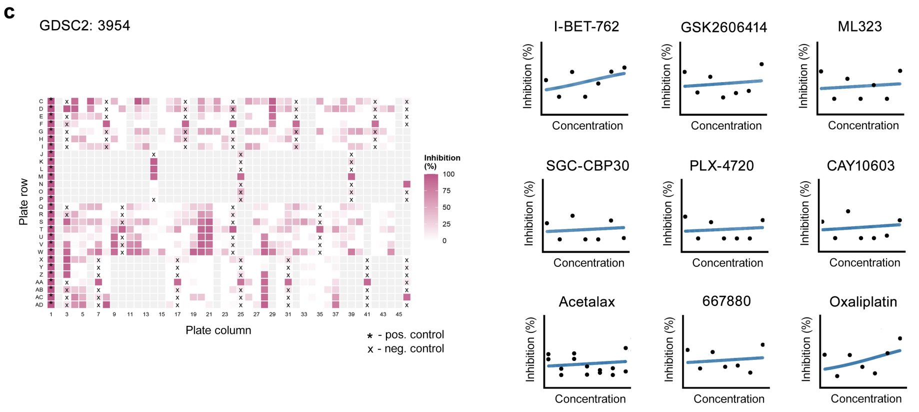

Metric	Value
Z-prime	0.5
SSMD	6
S/B ratio	40.2
NRFE	**14.1**

d

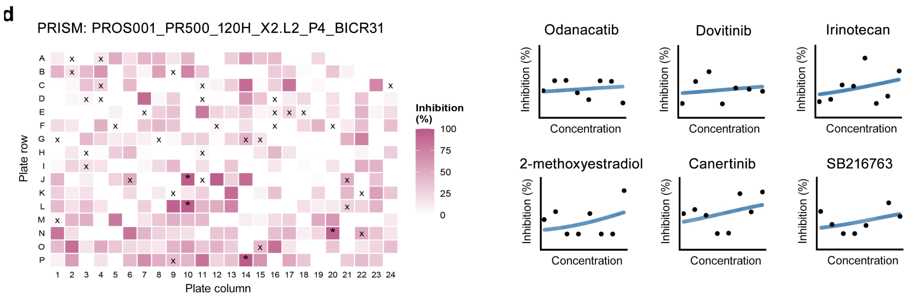

Metric	Value
Z-prime	0.7
SSMD	12
S/B ratio	***0**
NRFE	**21.8**

Supplementary Figure 2 | NRFE identifies systematic artefacts in drug screening datasets not detected by the standard metrics. (**a**) An example plate from GDSC1 dataset showing column-wise striping in the right half (left), which affects dose-response patterns of compounds with irregular dose-responses (middle). This systematic pattern was detected only by NRFE and Z-prime metrics (right, bold). (**b**) Another GDCS1 plate shows technical artefacts with potential precipitation/solubility issues (left), illustrated by sporadic drug response variations across concentrations of drug responses (middle), and identified solely by the NRFE metric (right). (**c**) An example of systematic dispensing artefacts in a drug plate from GDSC2 dataset (left), visible as abnormal variations specifically in the first and third drug doses across multiple compounds, which results in irregular jumpy behavior at these concentrations in the dose-response curves (middle), and was detected uniquely through the NRFE analysis (right). (**d**) An example of the plate from PRISM dataset

demonstrates a checkerboard-like pattern of alternating high- and low-value responses (left), reflecting potential systematic dispensing or temperature gradient effects, visible as alternating intensity fluctuations in dose-response profiles (middle), and identified by the NRFE analysis and S/B ratio (right).

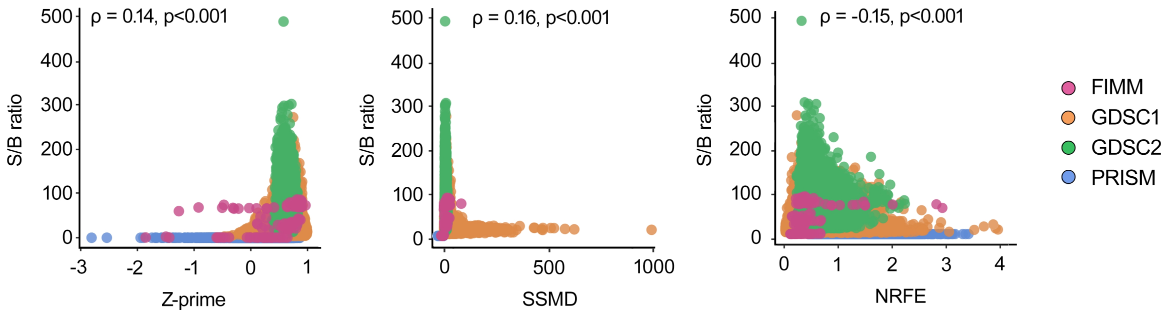

Supplementary Figure 3 | Correlation analysis between signal-to-background (S/B) ratio and the other quality metrics including Z-prime (left), SSMD (middle), and NRFE (right). Spearman correlations (ρ) are shown in each plot, with p < 0.001 for all comparisons, based on the permutation test.
